# Prevalence of acromegaly in patients with symptoms of sleep apnea

**DOI:** 10.1371/journal.pone.0183539

**Published:** 2017-09-12

**Authors:** Gemma Sesmilo, Eugenia Resmini, Marcel Sambo, Concepción Blanco, Fernando Calvo, Fernando Pazos, Pablo Fernández-Catalina, Purificación Martínez de Icaya, Concepción Páramo, Carmen Fajardo, Mónica Marazuela, Cristina Álvarez-Escolá, Juan Jose Díez, Verónica Perea, on behalf of the ACROSAHS study group

**Affiliations:** 1 Servicio de endocrinología, Hospital Quirón-Dexeus/Clínica del Sueño Estivill, Barcelona, Spain; 2 IIB- Sant Pau y Servicio de Endocrinología, Departamento de Medicina, Centro de Investigación Biomédica en Enfermedades Raras (CIBER-ER Unidad 747), Hospital Sant Pau, Universitat Autònoma de Barcelona, Barcelona, Spain; 3 Servicio de endocrinología, Hospital Universitario Gregorio Marañón, Madrid, Spain; 4 Servicio de endocrinología, Hospital Universitario Príncipe de Asturias, Madrid, Spain; 5 Servicio de endocrinología, Hospital Clínico Universitario Lozano-Blesa, Zaragoza, Spain; 6 Servicio de endocrinología, Hospital Universitario Marqués de Valdecilla, Santander, Spain; 7 Servicio de endocrinología, Complexo Hospitalario de Pontevedra, Pontevedra, Spain; 8 Servicio de endocrinología, Hospital Universitario Severo Ochoa, Leganés, Madrid, Spain; 9 Servicio de endocrinología, Complexo Hospitalario Universitario De Vigo, Vigo, Spain; 10 Servicio de endocrinología, Hospital Universitario de la Ribera, Alzira, Spain; 11 Servicio de endocrinología, Hospital de La Princesa, Madrid, Spain; 12 Servicio de endocrinología, Hospital Universitario la Paz, Madrid, Spain; 13 Servicio de endocrinología, Hospital Universitario Ramón y Cajal, Madrid, Spain; Charité - Universitätsmedizin Berlin, GERMANY

## Abstract

Acromegaly is a rare disease with nonspecific symptoms with acral enlargement being almost universally present at diagnosis. The estimated prevalence is 40–125 cases/million but targeted universal screening studies have found a higher prevalence (about 10 fold). The aim of the ACROSAHS study was to investigate the prevalence of acromegaly and acromegaly comorbidities in patients with sleep apnea symptoms and acral enlargement. ACROSAHS was a Spanish prospective non-interventional epidemiological study in 13 Hospital sleep referral units. Facial and acral enlargement symptoms including: ring size and shoe size increase, tongue, lips and jaws enlargement, paresthesia or carpal tunnel syndrome and widening of tooth spaces, as well as other typical acromegaly comorbidities were recorded with a self-administered questionnaire of patients who attended a first visit for sleep apnea symptoms between 09/2013 and 07/2014. Serum insulin-like growth factor type 1 (IGF1) was measured in patients with ≥1 acral symptom to determine the prevalence of acromegaly. Of the 1557 patients enrolled, 1477 with complete data (72% male) were analyzed. 530 patients (36%) reported at least 1 acral enlargement symptom and were tested for IGF-1, 41 were above range, persisted in 7, and among those, 2 cases of acromegaly were diagnosed (prevalence of at least 1.35 cases/1000). Overall, 1019 patients (69%) had ≥2 acromegaly symptoms and should have been screened according to guidelines; moreover 373 patients (25%) had ≥1 symptom of acral enlargement plus ≥3 other acromegaly symptoms. In conclusion, in patients with sleep apnea symptoms and acral enlargement, we found an acromegaly prevalence of at least 1.35 cases per 1000 and a high prevalence of typical acromegaly symptoms. It is important that sleep specialists are aware of acromegaly symptoms to aid with acromegaly diagnosis.

## Introduction

Acromegaly is a rare disease resulting from overproduction of growth hormone (GH) by the pituitary, mostly from a GH-producing adenoma [1]. It is an insidious condition that usually takes years of symptoms before a correct diagnosis, partly due to the high population prevalence of comorbidities associated with the disease and the low awareness of the condition. It is estimated that the diagnosis is delayed between 6 and 20 years after the appearance of the first symptom [2] and is thought to be under-recognized and under-diagnosed [3].

The estimated prevalence is 40 to 125 cases per million according to different epidemiological studies [4–8], but recent population based studies have shown that prevalence could be 5 to 15 times higher [9–13]. Schneider et al, in an unselected German population from a primary care center, found a prevalence of 1000 cases per million (1/1000) [9]. Rosario et al, in a Brazilian primary care population selected for symptoms of increased ring size or shoe size found a prevalence of 365 per million (0.365/1000) [10]. Rosario et al, in a selected population of patients with type 2 diabetes, found a prevalence of 480 cases per million (0.48/1000) [11]. The same authors showed that the probability of acromegaly in a hypertensive population without acral enlargement was very unlikely [12]. Recently, Galerneau et al described the prevalence of acromegaly in unselected patients with suspected sleep apnea-hypopnea syndrome (SAHS) and fount it to be 2.5/1000 [13].

Facial and acral enlargement is the most common symptom of the disease (70–80%), and it is highly prevalent at diagnosis [3,14,15]. Respiratory problems are major comorbidities responsible for 25% of the mortality in these patients [16]. SAHS has been reported in about 88% of patients with acromegaly (both active and inactive) [17,18] due to anatomical changes in bone structures and soft tissue enlargement of the upper airway [18]. These changes lead to snoring as a characteristic symptom of the disease.

Since there are not pathognomonic clinical features that allow for an early diagnosis, the American Association of Clinical Endocrinologists (AACE) guidelines [19] recommend (Grade A evidence) actively searching for the disease in patients with at least two of the following signs or symptoms: abnormal acral enlargement (hands, feet, facial changes) hypertension, diabetes, joint pain, fatigue, cardiac disorders including ventricular hypertrophy and systolic or diastolic dysfunction, headaches, carpal tunnel syndrome, sleep apnea syndrome, sweating, loss of vision, colonic polyps, and progressive mandibular malocclusion [19,20]. In recent guidelines [21], Katznelson et al recommended measuring insulin-like growth factor 1 (IGF-1) in patients who have several of these associated conditions: sleep apnea syndrome, type 2 diabetes mellitus, debilitating arthritis, carpal tunnel syndrome, hyperhidrosis, and hypertension. The prevalence of acromegaly in patients presenting sleep apnea symptoms has only been reported in one recent French study [13].

The aim of the present study was to investigate acromegaly prevalence in patients who are referred to sleep units for suspected sleep apnea syndrome and who also have symptoms of facial and acral enlargement. The secondary aim was to study the prevalence of typical acromegaly symptoms and comorbidities in order to estimate what proportion of patients should be screened according to current consensus guidelines. We also compared symptom prevalence between patients with persistently elevated IGF 1 and patients without elevated IGF-1.

## Patients and methods

This was an investigator initiated, multicenter, prospective epidemiological study, involving 13 Spanish hospitals. The protocol was reviewed and approved by the reference Ethics Committee as well as the local Ethics Committees. Signed informed consent was obtained from all patients and the reported investigations were carried out in accordance with the principles of the Declaration of Helsinki as revised in 2008.

New patients seen between September 2013 and July 2014 in the sleep referral units of the participating centers were offered to enter the study. To participate, patients had to have snoring or suspected SAHS, be over 18 years of age, and sign the informed consent. Exclusion criteria included: uncontrolled gastrointestinal or malabsorptive disease, pregnancy, severe liver or kidney disease (glomerular filtration rate [GFR] <30), and active cancer.

Patients followed the usual protocol evaluation for suspected SAHS at each referral unit and had a polysomnography (PSG) or conventional polygraphy when indicated. Test results as well as the apnea-hypopnea index (AHI) (number of apnea/hypopnea events per hour of recording), were collected in addition to the reasons for referral to the sleep unit. Enrolled patients underwent a complete physical examination including blood pressure, weight and height, and measurement of ring finger circumference and length (fourth finger of the non-dominant hand).

A self-administered questionnaire of symptoms and comorbidities typically found in acromegaly was provided to patients and was later discussed with the attending physician to resolve or clarify any possible doubts. Collected symptoms were divided in two groups: 1: those related to facial and acral enlargement: increased ring size, increased shoe size, lips or jaw enlargement, tooth space widening, tongue enlargement, paresthesia in hands or carpal tunnel syndrome and 2: other typical acromegaly symptoms and comorbidities including: headaches, hoarse voice, arthralgia, menstrual disturbances in women, erectile dysfunction in men, diabetes, high blood pressure, fatigue, and excessive perspiration. Patients who presented any symptom of the acral enlargement group underwent a blood draw for serum IGF-1 testing.

IGF-1 was determined at each center’s local laboratory. If the IGF-1 values were elevated, a second sample was obtained to repeat the analysis. In patients with re-confirmed supranormal IGF-1 (>2 standard deviations [SD] adjusted for age and sex), an oral glucose tolerance test (OGTT) with GH measurements was performed. GH was measured also at each of the collaborating centers. Nadir GH >0.4 ng/mL was considered abnormal. In patients with no GH suppression or high suspicion of acromegaly, a pituitary magnetic resonance imaging (MRI) was obtained.

Mean and standard deviation are reported for continuous variables, and number and percentage are reported for categorical variables. The statistical analysis was performed using R v3.02. The potential relationship between the response variable (presence of acromegaly and/or persistent IGF-1) and the explanatory variables (all collected secondary variables) was examined by means of bivariate analyses, applying the following methods: Chi-Square test, Fisher exact test or LR Chi-Square test for qualitative variables and Analysis of Variance or Kruskal-Wallis test for quantitative variables. The compliance of application conditions was performed using Shapiro-Wilk and Kolmogorov-Smirnov normality tests and Levene tests for homogeneity of variances. For all statistical tests a nominal significance level of 5% (P <0.05) was applied.

## Results

A total of 1557 patients were enrolled in the study. Eighty patients were excluded due to protocol violations (mainly presence of acral enlargement without IGF-1 results). A total of 1477 patients were analyzed, and their patient characteristics and demographic data are shown in Table 1.

**Table 1:** Patient demographics (N = 1477).

**Age *(years)***	55.6 ± 12.4
**Sex: male/female *(n*, *%)***	1064, 72%/413, 28%
**Weight *(Kg)***	90.2 ± 18.9
**Height *(cm)***	169 ± 9.7
**BMI**	31.7 ± 6.3
**Waist *(cm)***	108 ±15
**SBP *(mmHg)***	133 ±18.7
**DBP *(mmHg)***	79.9 ±13.7

BMI, body mass index; DBP, diastolic blood pressure; SBP, systolic blood pressure. Results are mean ± SD or % when indicated

Reasons for being referred to the sleep unit were: snoring (46%), day-time sleepiness (32%) and/or apnea-hypopnea (55%). Eighty-eight percent of patients (n = 1306) had available results for specific SAHS tests: polysomnography (37%) or conventional polygraphy (63%). Ninety-four percent of them were confirmed to have SAHS.

### Acromegaly prevalence

A total of 530 patients had IGF-1 measured and 41 were above the age- and sex-adjusted reference range. IGF-1 elevation in a second sample was reconfirmed in 7 patients. Only one of them had all pre-specified criteria for acromegaly diagnosis: IGF-1 persistently elevated, GH nadir >0.4 ng/mL and a pituitary microadenoma. A second patient with persistently high IGF-1 (2.8 times the upper limit of normal [ULN] of the gender and age range) had GH suppression on the OGTT (0.31 ng/mL) but presented a florid symptomatic acromegaly. Pituitary MRI showed a microadenoma. In both cases the tumor was removed and histologically proved with the subsequent IGF-1 normalization and amelioration of symptoms. Therefore, we found 2 proven cases of acromegaly in our cohort, which represents an acromegaly prevalence of at least 1354 cases per million (1.35/1000).

The other five cases of patients with persistently elevated IGF-1 (between 1.13 and 1.57 x ULN) had GH suppression on the OGTT (below 0.4 ng/mL). One of them had a 2.8 mm microadenoma on the MRI. All patients with persistently elevated IGF-1 are being followed.

### Acromegaly symptom prevalence

**Fig 1:**
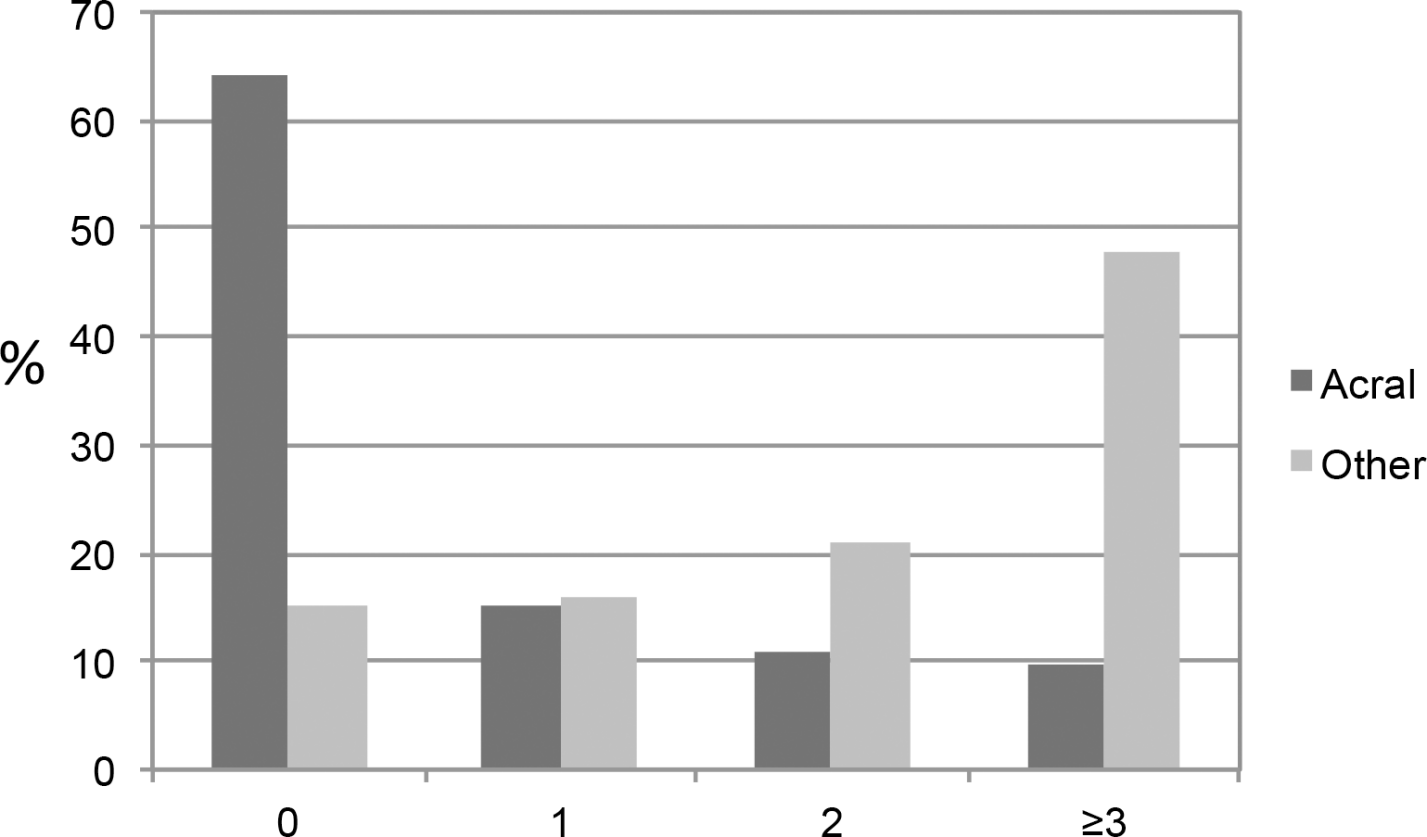
Number of acromegaly symptoms. Bars represent percent of patients with different number of acromegaly symptoms. Acral: acral enlargement symptoms. Other: other acromegaly symptoms.

Acral enlargement prevalence is shown in Table 2. A total of 530 patients (35.9%) reported at least one acral enlargement symptom: 226 (15.3%) had 1, 159 (10.8%) had 2 and 145 (9.8%) had ≥3 symptoms (Fig 1). The most common was paresthesia in hands (25.7%) followed by increase in ring size (17.6%) and increase in shoe size (10.8%).

**Table 2:** Acromegaly symptoms in the whole cohort.

**Acral enlargement symptoms**	**N = 1477** **n (%)**
Increased ring size	260 (17.6)
Increased shoe size	159 (10.8)
Lips or jaw enlargement	87 (5.9)
Tooth space widening	112 (7.6)
Tongue enlargement	55 (3.7)
Paresthesia in hands	380 (25.7)
**Other typical acromegaly symptoms**	
Headaches	355 (24.0)
Hoarse voice	261 (17.7)
Arthralgia	706 (47.6)
Menstrual disturbances (n = 273)	61 (4.1)
Erectile dysfunction (n = 930)	237 (16.0)
Diabetes	226 (15.3)
High blood pressure	630 (42.7)
Fatigue	810 (54.8)
Excessive perspiration	442 (29.9)

Prevalence of other typical acromegaly symptoms and comorbidities are shown in Table 2. Only 222 patients (15.0%) did not report to have any acromegaly symptoms or comorbidities, 236 (16.0%) reported 1, 310 (21.0%) 2 and 709 (48.0%) ≥3 acromegaly symptoms or comorbidities (Fig 1). The mean number of acromegaly symptoms and comorbidities in the whole cohort was 2.5 ± 1.75. Moreover, a total of 373 patients (25.3%) had ≥1 acral symptom and ≥3 other acromegaly symptoms.

### Clinical differences between patients with acromegaly and the rest

We compared the clinical characteristics of the patients with confirmed acromegaly (n = 2) with the rest. (n = 1475).

Apnea-hypopnea index (AHI) was significantly higher in patients with acromegaly: 81 (range: 72–90) vs 33 events per hour (range 18–52) (p = 0.045). Patients with acromegaly had a higher prevalence of hoarse voice (100% vs 19%). Moreover both patients with acromegaly had acral enlargement symptoms (100% vs 41%) and ≥3 other acromegaly symptoms (100% vs 60.5%). The total number of acral symptoms was 1.5 ± 1.1 in patients versus 0.71 ± 1.1 (p = ns). The total number of other acromegaly symptoms was 4.0 ± 1.4 in patients versus 2.5 ± 1.7 (p = ns).

### Clinical characteristics of patients with persistently elevated IGF-1

**Fig 2:**
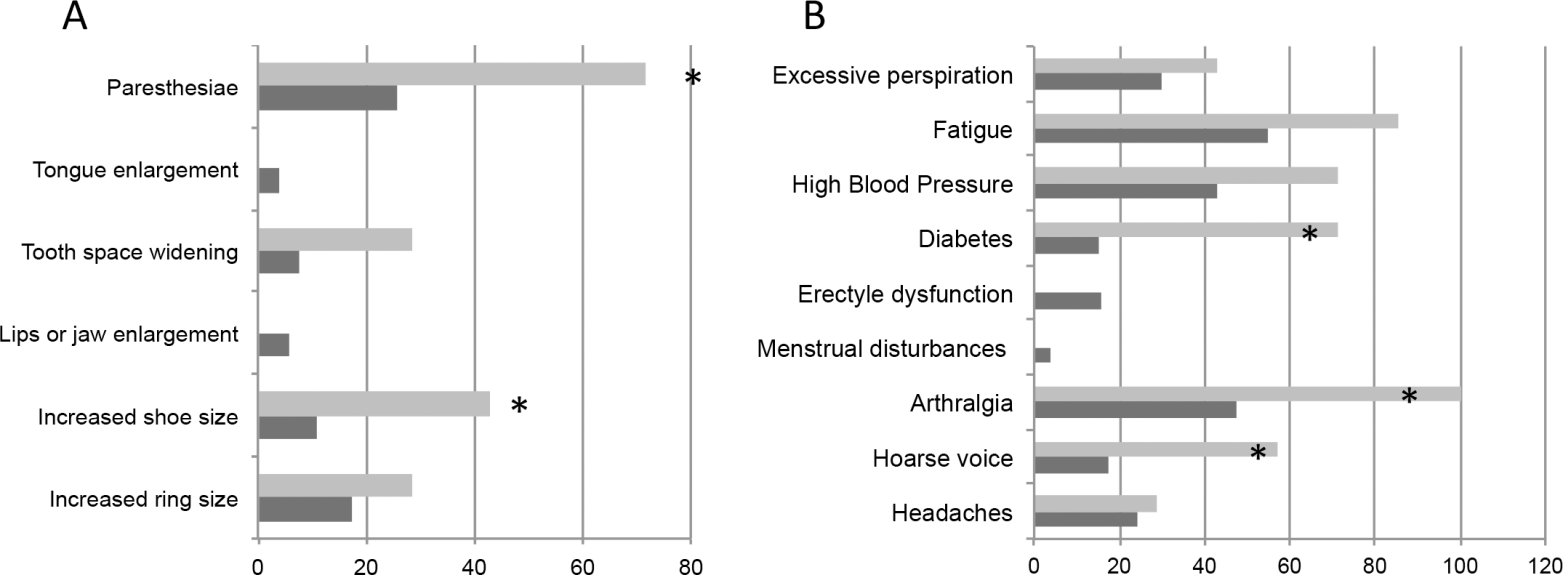
Prevalence of acromegaly symptoms. (A) acral enlargement and (B) other acromegaly symptoms in patients with persistently elevated IGF-1 (n = 7, light grey) and the rest of patients (n = 1470, dark grey). *p>0.05.

We analyzed the clinical characteristics of the 7 patients with persistently elevated IGF-1 (5 without a confirmed diagnosis of acromegaly and 2 with confirmed diagnosis) and compared them with the rest of the patients (n = 1470). Clinical characteristics are shown in Table 3. Symptom rates are shown in Fig 2. There was a higher prevalence of shoe size increase: (43% vs 11%; p = 0.027) paresthesia (71% vs 28%; p = 0.023), hoarse voice (57 vs 18%; p = 0.021) arthralgia (100% vs 47%; p = 0.005) and diabetes (71% vs 15%; p = 0.001) in patients with persistently elevated IGF-1. The total number of acral symptoms was 1.7 ± 0.9 versus 0.7 ± 1.1 (p = 0.03) and the total number of other acromegaly symptoms was 4.6 ± 0.9 versus 2.5 ± 1.7 (p = 0.001). Of note, all 7 patients (100%) had acral symptoms plus ≥3 acromegaly symptoms compared with 25% of the rest of the patients.

**Table 3:** Clinical and biochemical characteristics of the 7 patients with persistently elevated IGF1.

IGF1	Repeated IGF1	Mean IGF1	ULN	GH nadir	IGF1 x ULN	Sex	Age	MRI	Size^a^ (mm)
268	238	253	185	0.16	1.37	M	55	microadenoma	2.8
240	226	233	185	0.37	1.26	M	66	normal	
292	219	255.5	163	0.23	1.57	M	48	normal	
271	315	293	242	0.29	1.21	F	59	normal	
202	217	209.5	185	0.38	1.13	M	55	normal	
**Confirmed acromegaly**									
262	260	261	231	1.5	1.13	M	65	microadenoma	9
491	567	529	200	0.31	2.65	F	67	microadenoma	5

^a^Size: maximum diameter.

IGF1 and GH are in ng/mL. F, female; GH, growth hormone; IGF1, insulin-like growth factor 1; M, male; MRI, magnetic resonance imaging; ULN, upper limit of normal.

## Discussion

In the present study we have found a prevalence of acromegaly that was higher than expected in patients consulting to sleep referral units for sleep apnea symptoms and/or snoring that also had acral growth symptoms. The prevalence of ≥1.35 in 1000 cases is higher than what is estimated in the general population (0.04–0.12/1000) and it is closer to the prevalence reported by Schneider in an unselected population from primary care units (1/1000) [4–12] but lower than the prevalence recently reported by Galerneau et al in a non-selected population of patients with suspected SAHS (2.5/1000) [13]. In addition, our study reported a high prevalence of typical acromegaly symptoms and co-morbidities in the studied population: 36% of participants experienced symptoms of acral enlargement, with paresthesia being the most common acral symptom (29%), 69% had ≥2 other typical acromegaly symptoms and 48% had ≥3.

We found an acromegaly prevalence of at least 1.35 cases per 1000. In calculating this figure we have assumed that there were no cases in the group who did not refer acral changes, but we cannot exclude the possibility of acromegaly in that group. Moreover, we have not included the 5 patients with persistently elevated IGF-1 with GH suppression in the OGTT. Although we cannot completely rule out acromegaly in those 5 cases, all IGF-1 elevations were mild and there was no tumor in 4 of the patients and the physicians who cared for those patients decided to follow-up on them with no specific treatment. In the selected population of patients with suspected SAHS, acromegaly prevalence is at least 10 to 20 times higher than what is assumed for the general population [4–8].

Of note, one of the acromegaly cases, despite having a clearly elevated IGF-1 (2.8 x ULN), had GH suppression by glucose (nadir GH: 0.31 ng/mL). The patient also had diabetes and grade II obesity (BMI = 35). Several investigators have reported that acromegaly may be present despite GH suppression on the OGTT [22–25]. Dimaraki et al, found that 50% of acromegaly cases occurred with GH suppression in a series of patients referred to a specialized unit, for reasons including discrepancies in diagnostic tests [22]; however, they estimated that in the general acromegaly population, that figure is probably close to 25%. Freda hypothesized that the discrepancies may depend on the GH assay used and the cut-off considered [25]. In explaining discrepancies, we have to account that 85% of somatotropinomas are macroadenomas; it is possible that microtumors could still respond to glucose, similar to what happens in other endocrine adenomas (parathyroid adenomas responding to calcium or adrenocorticotropic hormone producing tumors responding to high doses of dexamethasone). In the present study, the patient with GH suppression on the OGTT had a small microtumor only seen in the 3-tesla pituitary MRI (5 mm maximum diameter).

Remarkably, patients with persistently elevated IGF-1 had a higher prevalence of typical acromegaly symptoms and comorbidities (100% had ≥3), and the number of acral as well as other acromegaly symptoms was significantly higher than in the rest of population studied. We cannot completely rule out acromegaly in the group of patients with persistently elevated IGF-1, even though 4 of 5 patients had a normal MRI (and one had a normal octreoscan to rule out an ectopic source), but there have been described acromegaly cases without a visible adenoma in the MRI [26]. If the 5 cases with persistently elevated IGF-1 in our study had been considered as having acromegaly, we would be facing a very high prevalence of the disease in this particular population.

Similar to our results, Galerneau et al found 6 patients with persistently elevated IGF-1 and normal GH on the OGTT in a cohort of 817 patients with suspected SAHS [13].

In patients with elevated IGF-1 levels and normal GH suppression, one should also consider accuracy of the IGF-1 measurement. It is known that different IGF-1 assays perform differently and that in a UK study, the diagnosis of acromegaly was inaccurately excluded in 30% of single samples assayed for IGF-1 in 23 different laboratories [27]. In our study, there were 34 patients with a high IGF-1 value that was not reconfirmed in a second sample. Since this was a screening study, each of the 13 centers used their standard IGF-1 assay in order to reproduce “real life” screening conditions.

We found a high prevalence of typical acromegaly symptoms in the studied population. According to AACE guidelines, 69% of the patients (who had ≥2 typical acromegaly symptoms or comorbidities) should have undergone a screen test for acromegaly [19]. This result is very similar to what has been recently reported by Galerneau et al [13]. Current guidelines suggest screening if several acromegaly symptoms are present [21]; therefore, a high number of patients who consult to rule out sleep apnea syndrome would need an IGF-1 test to rule out GH excess. Since acral enlargement is almost universally present in acromegaly [3,14,15], recognizing acral enlargement features would lower the population requiring screening to 36%. We believe that it is necessary to train specialists who care for these patients on the importance of recognizing acral enlargement features. Moreover, the two acromegaly cases (as well as the other 5 patients with persistently high IGF-1), had at least one acral symptom and three or more other typical acromegaly symptoms. A 25.5% of the studied population met those criteria; we believe at least that subgroup should be screened. Economic studies to prove cost-effectiveness are warranted. Since symptoms attributed to SAHS may improve with SAHS treatment, it may be interesting to see if after treatment, the population to be screened narrows down.

Acromegaly symptoms, even acral enlargement, are non-specific. Patients with SAHS are usually overweight or obese [28], mean BMI of the cohort was in the obesity range -31.6 kg/m^2^-, it is possible that obesity played a confounding role in the evaluation of symptoms in this patient cohort. Regarding physiopathology, acromegaly may cause sleep apnea due to facial anatomical changes and soft tissue enlargement [18]. The prevalence of SAHS in patients with acromegaly is very high according to several studies (60–88%) [17–18, 29]. Acromegaly causes macroglosia and upper airway narrowing due to laryngeal soft tissue enlargement [18]. Among the different cephalometric measurements taken on the MRI, enlarged soft palate, uvula and tongue are considered directly related to SAHS pathogenesis [18, 30]. Treatment of acromegaly both with somatostatin analogs and pegvisomant has been shown to improve SAHS severity but there are variable responses and up to 40–50% of controlled patients may still have SAHS [31, 32]. An early diagnosis of the disease may help in avoiding comorbidities.

Limitations to this study have to be acknowledged. Even though acral enlargement is almost universally present at diagnosis in acromegaly [3,14,15], it is known that sometimes patients do not recognize slow developing symptoms on themselves. This could have underestimated our results; however, the prevalence of acromegaly in our study would still be at least 1.35/1000. One could also argue that the 5 cases with persistently elevated IGF-1, particularly the one with a pituitary microadenoma are in fact, acromegaly cases. We did not include them in the acromegaly group because we did not have pathological certainty of an acromegaly diagnosis and the findings could have been related to the IGF-1 assay. However, patients with persistently elevated IGF-1 clearly had a high prevalence of several acromegaly symptoms and comorbidities. At the moment, we have chosen to be cautious and are closely following these 5 patients before making a specific diagnosis.

## Conclusions

In conclusion, we have found an acromegaly prevalence of at least 1.35 cases per 1000 in a population of patients with sleep apnea symptoms and acral enlargement who consult to a sleep specialist. We have also found a very high prevalence of typical acromegaly symptoms in this cohort. It is important that sleep specialists are aware of the disease and recognize its symptoms, particularly those related to facial and acral enlargement, in order to perform an early diagnosis.
